# Comprehensive Analyses of Ferroptosis-Related Alterations and Their Prognostic Significance in Glioblastoma

**DOI:** 10.3389/fmolb.2022.904098

**Published:** 2022-06-03

**Authors:** Yuan Tian, Hongtao Liu, Caiqing Zhang, Wei Liu, Tong Wu, Xiaowei Yang, Junyan Zhao, Yuping Sun

**Affiliations:** ^1^ Somatic Radiotherapy Department, Shandong Second Provincial General Hospital, Jinan, China; ^2^ Department of Pathology, Shandong Medicine and Health Key Laboratory of Clinical Pathology, The First Affiliated Hospital of Shandong First Medical University and Shandong Provincial Qianfoshan Hospital, Shandong Lung Cancer Institute, Shandong Institute of Nephrology, Jinan, China; ^3^ Department of Respiratory and Critical Care Medicine, Shandong Second Provincial General Hospital, Shandong University, Jinan, China; ^4^ Department of Hepatobiliary Intervention, Beijing Tsinghua Changgung Hospital, School of Clinical Medicine, Tsinghua University, Beijing, China; ^5^ Nursing Department, The First Affiliated Hospital of Shandong First Medical University & Shandong Provincial Qianfoshan Hospital, Jinan, China; ^6^ Phase I Clinical Trial Center, Shandong Cancer Hospital and Institute, Shandong First Medical University and Shandong Academy of Medical Sciences, Jinan, China

**Keywords:** ferroptosis, alterations, predictive models, prognosis, glioblastoma

## Abstract

**Background:** This study was designed to explore the implications of ferroptosis-related alterations in glioblastoma patients.

**Method:** After obtaining the data sets CGGA325, CGGA623, TCGA-GBM, and GSE83300 online, extensive analysis and mutual verification were performed using R language-based analytic technology, followed by further immunohistochemistry staining verification utilizing clinical pathological tissues.

**Results:** The analysis revealed a substantial difference in the expression of ferroptosis-related genes between malignant and paracancerous samples, which was compatible with immunohistochemistry staining results from clinicopathological samples. Three distinct clustering studies were run sequentially on these data. All of the findings were consistent and had a high prediction value for glioblastoma. Then, the risk score predicting model containing 23 genes (*CP, EMP1, AKR1C1, FMOD, MYBPH, IFI30, SRPX2, PDLIM1, MMP19, SPOCD1, FCGBP, NAMPT, SLC11A1, S100A10, TNC, CSMD3, ATP1A2, CUX2, GALNT9, TNFAIP6, C15orf48, WSCD2*, and *CBLN1*) on the basis of “Ferroptosis.gene.cluster” was constructed. In the subsequent correlation analysis of clinical characteristics, tumor mutation burden, HRD, neoantigen burden and chromosomal instability, mRNAsi, TIDE, and GDSC, all the results indicated that the risk score model might have a better predictive efficiency.

**Conclusion:** In glioblastoma, there were a large number of abnormal ferroptosis-related alterations, which were significant for the prognosis of patients. The risk score-predicting model integrating 23 genes would have a higher predictive value.

## 1 Introduction

Ferroptosis, a kind of controlled cell death triggered by excessive lipid peroxidation (Jiang et al., 2021), has been implicated in tumor suppression mechanisms (Chen et al., 2021) and may play a critical role in carcinogenesis and precision medicine (Shen et al., 2018; Liang et al., 2019). Additionally, it has been implicated in the control of a variety of tumor-associated signaling pathways (Wu et al., 2019; Lee et al., 2020). CD8^+^ T lymphocytes have been shown to be able to modulate tumor ferroptosis in studies in the field of tumor immunotherapy (Wang et al., 2019). Ferroptosis-related reports have been published in the fields of chemotherapy, radiation, and immunotherapy, demonstrating its distinct potential for tumor treatment (Yee et al., 2020; Liu et al., 2022; Zhao et al., 2022).

With the advancement of bioinformatics analysis and sequencing technology, it is becoming increasingly convenient for us to analyze the genomic alterations associated with certain diseases using publicly available data (Qu et al., 2016; Chen et al., 2021). Reports utilizing online data to uncover important genetic abnormalities in glioblastoma are frequent (Rutledge et al., 2013; Cimino et al., 2018) but seldom use ferroptosis. This study was created using R-based bioinformatics analytic tools and publicly available gene data from Internet sources.

## 2 Materials and Methods

The flow diagram of the study is provided in Supplementary Figure S1. The specific details were listed as follows.

### 2.1 Data Download Collection

#### 2.1.1 TCGA-GBM Data Download

Gene expression data (https://tcga-xena-hub.s3.us-east-1.amazonaws.com/latest/TCGA.GBM.sampleMap%2FHiSeqV2.gz), genotype data (https://tcga-xena-hub.s3.us-east-1.amazonaws.com/latest/TCGA.GBM.sampleMap%2FGBM_clinicalMatrix), survival data of patient samples (https://tcga-xena-hub.s3.us-east-1.amazonaws.com/latest/survival%2FGBM_survival.txt.gz), TCGA-GBM mutant “maf” (mutation annotation format) file (https://portal.gdc.cancer.gov/files/da904cd3-79d7-4ae3-b6c0-e7127998b3e6), TCGA-GBM gene copy number data (https://gdc-hub.s3.us-east-1.amazonaws.com/latest/TCGA-GBM.gistic.tsv.gz), and the masked copy number segment file of TCGA-GBM were downloaded from the GDC database by the R package TCGAbiolinks (v 2.16.4).

#### 2.1.2 CGGA Data Download

The data of CGGA, including mRNAseq_693, mRNAseq_325, and mRNA sequencing data (non-glioma as control), were downloaded from the following link: http://www.cgga.org.cn/download.jsp.

#### 2.1.3 GSE83300 Data Download

The GSE83300 data were downloaded from the GEO database by the R package “GEOquery (v2.54.1).”

#### 2.1.4 The Genomic Damage Information of TCGA Samples Was Mainly Collected From PMID: 29617664 (Knijnenburg et al., 2018)

mutLoad_nonsilent (TMB): silent mutation load per Mb. CNA_frac_altered (CNV): fraction of genome altered (fraction of bps belonging to “altered” segments), where “altered” was defined as having relative CN >0.1 or <−0.1. HRD_Score: homologous recombination deficiency score calculated from three scores (TAI + LST + HRD_LOH). HRD_TAI: number of subchromosomal regions with allelic imbalance extending to the telomere. HRD_LST: number of chromosomal breaks between adjacent regions of at least 10 Mb. HRD_LOH: the number of LOH regions of intermediate size (>15 MB but < whole chromosome in length) (Knijnenburg et al., 2018).

#### 2.1.5 Ferroptosis-Involved Information

Ferroptosis was mainly derived from the FerrDb database (http://www.zhounan.org/ferrdb/) [(PMID: 32760210) and (PMID: 33330074)] (Liang et al., 2020; Zhuo et al., 2020). Then, the three were merged.

#### 2.1.6 MSigDB Data Download

MSigDB data (Molecular Signatures Database) were downloaded from the following link: https://www.gsea-msigdb.org/gsea/msigdb/index.jsp.

#### 2.1.7 Human (Gene Transfer Format) Files Download

The human “gtf” file (Homo_sapiens.GRCh38.99.gtf.gz) was downloaded from the Ensembl database, and then the symbol information was collected: (http://www.ensembl.org/info/data/ftp/index.html). The four data set samples were integrated, and the ComBat() function of the R package sva was used to remove the batch effect, and then subsequent analysis was performed.

### 2.2 Comprehensive Analysis of Ferroptosis-Related Genes

#### 2.2.1 Comparison of Ferroptosis-Related Gene Diseases and Normal Expression

The “non-glioma as control” data downloaded from the CGGA database were regarded as the normal sample, and then the difference in ferroptosis-involved gene expression between cancer and normal samples in the integrated data was compared; “wilcox.test()” was used to detect significant differences, and “ggpubr (v0.4.0)” was used to achieve visualization; **** means *p* < 0.0001, *** means *p* < 0.001, ** means *p* < 0.01, and * means *p* < 0.05.

#### 2.2.2 Mutation and Copy Number Variation Analysis

The gene-level CNV data of TCGA-GBM were downloaded, the copy number alterations of ferroptosis-related genes were counted, and then the variation frequency was calculated, and the R package ggplot2 (v 3.3.2) was used to draw the statistical graph.

#### 2.2.3 Circos Display and Principal Component Analysis of the Position on the Chromosome

From the human chromosome “gtf” file, the location information of the ferroptosis gene was extracted. Then, the R package RCircos (v1.2.1) was used to draw a gene circos map for position display. The function “prcomp()” of R was used to perform the principal component analysis (PCA) of cancer and normal samples. Then, R packages pca3d (v0.10.2) and rgl (v0.105.22) were adopted to draw the 3D version of the PCA.

### 2.3 Ferroptosis-Involved Cluster Analysis

#### 2.3.1 Cluster Analysis of Ferroptosis-Involved Genes

Based on the expression data of ferroptosis-involved genes in cancer samples, the package ConsensusClusterPlus (v1.50.0) was used to perform the unsupervised clustering of ferroptosis genes. The clustering algorithm used was k-means. Then, combined with the overall survival (OS) data, the R packages including survival (v3.2-7) and survminer (v0.4.8) were used to perform univariate Cox analysis on all ferroptosis-involved genes, and the differences and expression correlations (Pearson coefficient) among every ferroptosis genes would be calculated. Finally, the aforementioned results would be visualized by the Cytoscape (v3.7.2).

#### 2.3.2 Unsupervised Cluster Analysis of Samples

Based on the expression data of ferroptosis-related genes, the R package ConsensusClusterPlus (v1.50.0) was used to perform the unsupervised clustering of cancer samples. The clustering algorithm used was “pam,” and the distance used was “pearson.” Then, the R packages of survival and survminer were used to analyze the survival of the obtained subtypes, and then the Kaplan–Meier curve would be drawn.

### 2.4 Gene Set Variation Analysis Function Enrichment Analyses

Using the R package GSVA (v1.34.0), based on the KEGG data in MSigDB, functional enrichment analysis were performed on the samples, and then “limma” (v3.42.2) was used to retrieve the differential enrichment entries among subtypes, and the relevant threshold was set as “adj.p.value<0.05 & | logFC|> 0.3”; Finally, the R package ComplexHeatmap (v2.2.0) was used to draw the heatmap for visualization.

### 2.5 The Proportion and Difference of Immune-Infiltrating Cells in Different Ferroptosis Clusters Evaluated by Single Sample Gene Set Enrichment Analysis

The R package “GSVA” was used to calculate the enrichment score of 28 immune-infiltrating cells in cancer samples. After the results were obtained, the data would be normalized by the “scale()” function. According to the formula “(x-min(x))/(max(x)-min(x),” the data would be distributed from 0 to 1, and then the “wilcox.test()” was used to evaluate the significance of the difference in the proportion of immune cells among different ferroptosis cluster samples. “ggpubr” was used to achieve visualization. **** means *p* < 0.0001, *** means *p* < 0.001, **means *p* < 0.01, and *means *p* < 0.05. Finally, the Cox univariate regression analysis was performed on the proportion of immune cells, and *p* < 0.05 was used as the threshold to select immune cells that were significantly related to the prognosis.

### 2.6 Correlation Analysis Between Different Ferroptosis Clusters and Clinical Characteristics

The proportion of age, gender, chemo_therapy, and IDH1 mutations in different subtypes would be calculated, and relevant bar graphs would be drawn. Then, the “kruskal.test()” was used to test the significant difference in feature distributions among different subtypes.

### 2.7 Display of Ferroptosis-Related Genes in Different Ferroptosis Clusters

The expression differences of ferroptosis-related genes in different ferroptosis cluster subtypes would be counted, and then a heatmap for visualization would be drawn.

### 2.8 Screening of Differentially Expressed Genes in the Ferroptosis Cluster and Enrichment Analysis of DEGs

The R package “limma” was used to obtain DEGs among different subtypes. The threshold was set at “|logFC|> 1 and adj.p.val<0.05.” Afterward, the R package “clusterProfiler (v3.14.3)” was used for the functional enrichment analysis of DEGs. “*p* < 0.05” and “*q* < 0.2” were taken as the thresholds to filter the enrichment pathway, the enrichment factor would be calculated, and then the corresponding bubble chart would be drawn. The calculation formula of the enrichment factors is:

Enrichment factors = (the number of genes enriched into the pathway in the gene set)/(total number of genes in the pathway).

### 2.9 Ferroptosis.Gene.Cluster Obtained Based on the Cluster Analysis of DEGs

Through unsupervised clustering of samples based on DEGs, Ferroptosis.gene.cluster was obtained, and the heatmap of DEGs in different subtypes was drawn. Then, based on Ferroptosis.gene.cluster for survival analysis, the Kaplan–Meier survival curve was drawn.

### 2.10 Construction of “Ferroptosis Gene Signatures” Based on the DEGs of the “Ferroptosis Cluster”

First, univariate Cox regression analyses on the DEGs in different “ferroptosis clusters” were performed, and *p* < 0.05 was taken as the threshold to screen out genes that were significantly related to survival. Then, the R package “randomForest (v 4.614)” was used to perform random forest screening for survival significantly related genes. The related parameters used were “mtry = 2” and “ntree = 1,000.” Then, “MeanDecreaseGini> 0.72” was taken as the threshold to obtain key genes. Based on the expression of key genes, principal component analysis (PCA) on the sample was performed. The risk score is calculated by the following formula:

$$\text{R}\text{S}\text{c}\text{o}\text{r}{\text{e}}_{i}=\sum \left(PC1_{i}+PC2_{i}\right).$$ (1)

Among them, PC1 and PC2 represent the scores of principal component 1 and component 2, respectively, and “i” represents the corresponding sample.

### 2.11 The Evaluation of the Prognostic Efficacy

After the sample risk score was obtained, the high- and low-risk score groups were divided by the median node, the survival analysis was performed in the high- and low-risk score groups, and then the Kaplan–Meier survival curve was drawn. At the same time, it was verified in the internal sub-data sets of TCGA, CGGA (CGGA325/CGGA693), and GSE83300.

After that, the time-based ROC curve was further drawn, and the AUC values of 1, 3, and 5 years were all greater than 0.6, indicating that the predictive performance of the model was better. Finally, “ggalluvial (v0.12.3)” and “ggplot2” were used to draw Sankey diagrams to express the relationship of the data characteristics.

### 2.12 Correlation Analyses of Risk Score, “Ferroptosis Gene,” and Pathway Function

The correlation between the risk score and the differential enrichment pathway score (Pearson correlation coefficient) and the correlation between the “ferroptosis gene” and the differential enrichment pathway score (Pearson correlation coefficient) are further calculated, and the R package “corrplot (v 0.84)” was used to complete the visualization of relevance.

### 2.13 Difference Analysis of Risk Score in Different Groups

#### 2.13.1 Differences in Enrichment Scores Between High- and Low-Risk Score Groups

The differences in enrichment scores between the high- and low-risk score groups would be calculated, and the significance of the difference would be calculated by the R package “wilcox.test()” and visualized by “ggpubr.” **** means *p* < 0.0001, *** means *p* < 0.001, **means *p* < 0.01, and *means *p* < 0.05.

#### 2.13.2 Risk Score Differences in Different Ferroptosis.gene.clusters

The risk score differences in different Ferroptosis.gene.clusters would be counted. The significance of the difference would be calculated by the R package “wilcox.test()” and visualized by “ggpubr.” **** means *p* < 0.0001, *** means *p* < 0.001, ** means *p* < 0.01, and * means *p* < 0.05.

#### 2.13.3 Risk Score Differences in Different “Ferroptosis Clusters”

The risk score differences in different “ferroptosis cluster” would be counted. The significance of the difference would be calculated by the R package “wilcox.test()” and visualized by “ggpubr.” **** means *p* < 0.0001, *** means *p* < 0.001, ** means *p* < 0.01, and * means *p* < 0.05.

### 2.14 Risk Score Differences in Clinical Characteristics and Different Molecular Types

The distribution of risk score in age, gender, chemo_therapy, IDH1 mutation grouping, “ferroptosis cluster,” and “Ferroptosis.gene.cluster” was further checked. Then, the grouped box plot would be drawn, and the significant difference would be calculated by the R package “kruskal.test().” **** means *p* < 0.0001, *** means *p* < 0.001, ** means *p* < 0.01, and * means *p* < 0.05.

The correlation between risk score and mutation load, homologous recombination deficiency, neoantigen load, chromosomal instability (TMB, CNV, HRD, HRD_TAI, HRD_LST, HRD_LOH, DEL, INS, and SNP), and mRNAsi would be calculated. The linear correlation graph would be drawn.

### 2.15 The Landscape of the High- and Low-Risk Score Groups

The R package “TCGAbiolinks (v2.16.4)” was used for the GBM’s masked copy number segment data download, and the marker file data were downloaded from the GDC Reference File (https://gdc.cancer.gov/about-data/gdc-data- processing/gdc-reference-files); Then, “GenePattern GISTIC_2.0” (https://cloud.genepattern.org/gp/pages/index.jsf) was used to analyze the alterations of CNV in the two groups online, and finally, the R package “maftools (v1.0-2)” was used for visualization.

Based on the “maf” (mutation annotation format) file of GBM mutation and risk score grouping, the mutation landscape of the two sets would be drawn by the R package “maftools.”

### 2.16 Immunotherapy Analysis Results in the High- and Low-Risk Score Groups (TIDE Prediction + GDSC)

#### 2.16.1 Analysis Results of GDSC in the High- and Low-Risk Groups, Estimated by the IC_50_ Value

The R package “pRRophetic (v0.5)” was used to predict drug treatment response in the high- and low-risk score groups, and then the box plot describing the difference would be drawn. Significant differences among groups were tested by the R package “wilcox.test().” **** means *p* < 0.0001, *** means *p* < 0.001, ** means *p* < 0.01, and * means *p* < 0.05.

The TIDE score was used to predict the immunotherapy effect. The TIDE score could be obtained from the online website (http://tide.dfci.harvard.edu). Patients with higher TIDE scores enjoyed poorer therapeutic efficacy of immune checkpoint inhibitors and were related to the survival rate of those patients with worse anti-PD-1 and anti-CTLA-4 treatment results. After the TIDE score was obtained, the difference in different subtypes and risk groups would be calculated, and the significant difference would be tested by the R package “wilcox.test().” **** means *p* < 0.0001, *** means *p* < 0.001, ** means *p* < 0.01, and * means *p* < 0.05.

#### 2.16.2 Expression Differences of Immune Check Sites in the High- and Low-Risk Groups

The expression of immune checkpoints in the high- and low-risk groups would be further counted and drawn as a box-plot display; “wilcox.test()” was used to detect the significant difference. **** means *p* < 0.0001, *** means *p* < 0.001, ** means *p* < 0.01, and * means *p* < 0.05.

### 2.17 Immunohistochemical Validation Results of Clinical Samples

The glioblastoma samples used for this study were collected from the First Affiliated Hospital of Shandong First Medical University & Shandong Provincial Qianfoshan Hospital from June 2019 to February 2022 with informed consent provided by all participants. All tumor tissue specimens were surgically resected followed by formalin fixation and paraffin embedding (FFPE) for histological evaluation. All HE-stained and immunohistochemical (IHC)-stained slides were examined and confirmed to be glioblastoma by two experienced pathologists independently according to WHO criteria.

Slides were IHC-stained with specific primary antibodies (mouse anti-human p53 monoclonal antibody: cat. No. MAB-0674, clone MX008; mouse anti-human IDH1 R132H monoclonal antibody: cat. No. MAB-0733, clone MX031; mouse anti-human Ki-67 monoclonal antibody: cat. No. MAB-0672, clone MX006; mouse anti-human MGMT monoclonal antibody: cat. No. MAB-0361, clone MT3.1). All primary antibodies and secondary antibodies [sheep anti-mouse immunoglobulin G (IgG) polymer] were purchased from MXB Biotechnologies, Fuzhou, China. Slides were processed using an automated Roche BenchMark XT staining system according to the manufacturer’s protocol. Other genes (Ki-67, MGMT, and IDH1) were immunohistochemically stained in the same way.

## 3 Results

### 3.1 Data Collation

The flow diagram of the study is provided in Supplementary Figure S1. TCGA-GBM data were downloaded from UCSC Xena. After removing the samples without survival data, the expression matrix of 116 cancer samples was obtained. CGGA325 and CGGA693 were downloaded from the CGGA database, and then GBM data were extracted. After removing the samples without survival data, expression matrices of 137 and 237 samples were obtained, respectively. The GSE83300 data were downloaded from the GEO database; survival data and expression matrices of 50 samples were obtained. After integrating the three data sets and removing the batch effects, a total of 590 cancer samples were obtained for subsequent analyses.

Ferroptosis-related genes were downloaded from the database and two literatures (Liang et al., 2020; Zhuo et al., 2020), and finally 291 ferroptosis-related genes were obtained, of which 257 ferroptosis-related genes were displayed with expression information. The expression matrix of ferroptosis-related genes was extracted for subsequent analyses. Basic characteristics of all the data were provided in (Table 1).

**TABLE 1:** Basic characteristics of all the data.

	TCGA-GBM	CGGA325	CGGA693	GSE83300
Total	166	137	237	50
Age				
>60	81	14	52	4
≤60	85	123	185	46
Gender				
Male	107	87	139	25
Female	59	50	98	25
Chemo_therapy				
Yes	117	99	199	0
No	31	34	27	0
NA	18	4	11	50
IDH1_mut				
Mutant	6	39	45	0
Wild type	113	98	182	0
NA	47	0	10	50
Cluster				
cluster1	84	145	101	32
cluster2	53	92	65	18
Cluster.gene				
Cluster1	80	151	96	29
Cluster2	57	86	70	21
Event				
Dead	133	124	197	39
Alive	33	13	40	11

### 3.2 The Overall Display of Ferroptosis-Related Genes

#### 3.2.1 Expression Display of Ferroptosis-Related Genes in Diseases and Normal Samples

Twenty non-glioma data were downloaded from the CGGA database and taken as the normal control, and then the expression difference of ferroptosis-related genes between the cancer and normal samples in the integrated data was compared. Among them, 239 ferroptosis-related genes were found to be significantly different between cancer and normal samples (*p* < 0.05), indicating that most of the expression of ferroptosis-related genes were related to GBM (Supplementary Figure S2).

#### 3.2.2 The Mutations and CNV of Ferroptosis-Related Genes

**FIGURE 1:**
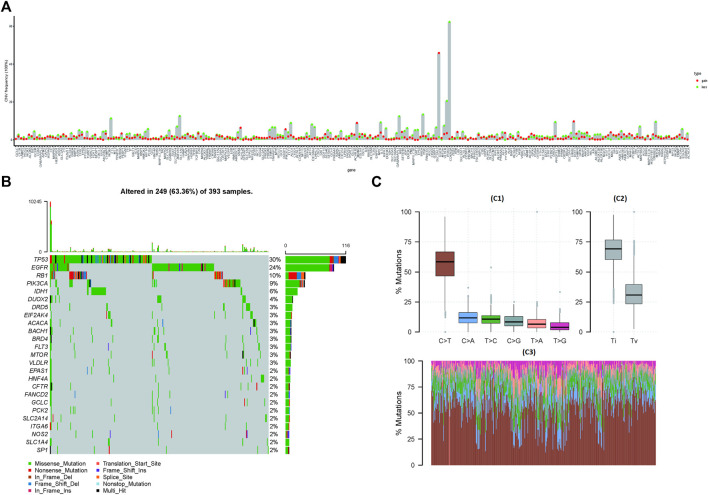
Mutations and copy number variations (CNVs) in ferroptosis-related genes. **(A)** CNV of ferroptosis-related genes. The abscissa axis represents the name of the related genes; the ordinate axis represents the CNV frequency. The type of CNV represented by red is gain; the type of CNV represented by green is loss. **(B)** Waterfall chart of ferroptosis-related gene mutations. The ordinate axis on the left represents the names of the top 25 genes, and the ordinate axis on the right represents the mutation frequency of the corresponding genes; different colors represent different types of gene alterations. **(C)** SNP of TCGA-GBM samples (C1): the abscissa axis represents the type of SNP; the ordinate axis represents the mutation percentage. (C2): the abscissa axis represents the type of variants (transitions or transversions); the ordinate axis represents the percentage of mutations. (C3): the abscissa axis represents the TCGA-GBM samples, in which different colors represent different SVP types, and the ordinate axis represents the percentage of variation in each sample.

The copy number data of ferroptosis-related genes was extracted from the gene-level copy number variation (CNV) data of TCGA-GBM (including 628 samples), and then the CNV map was drawn. Among them, CDKN2A deletion was found in more than 60% samples, and EGFR duplication was found in 40% samples (Figure 1A). The mutations of ferroptosis-related genes were further extracted from the TCGA-GBM “maf” file (containing 393 samples), and then the top 25 genes were selected and drawn into a waterfall chart, among which TP53 and EGFR ranked the top two mutation frequencies (>20%). The C > T variation in SNP was the most common (Figures 1B,C).

#### 3.2.3 Positional Circos Display on Chromosomes and Principal Component Analysis

The location information of ferroptosis-related genes was extracted from human chromosomal “gtf” files, and then a gene circos diagram was drawn for location display. Finally, the PCA results of the cancer and normal samples were drawn (Supplementary Figure S3A,B).

### 3.3 Ferroptosis-Related Cluster Analyses

#### 3.3.1 Cluster Analyses of Ferroptosis-Related Genes

Based on the expression information of ferroptosis-related genes in cancer samples, the unsupervised clustering of ferroptosis-related genes was performed, and three subtypes were obtained. After that, batch Cox univariate regression analyses were performed on ferroptosis-related genes, and the Pearson coefficients among different ferroptosis-related genes were calculated at the same time. Finally, the aforementioned results were visualized by Cytoscape (Supplementary Figure S3C).

#### 3.3.2 Unsupervised Cluster Analyses of Cancer Samples

Based on the expression information of ferroptosis-related genes, unsupervised cluster analyses of cancer samples were performed, and two cluster subtypes were obtained. After the survival analysis was performed on the two subtypes, the survival difference between the two subtypes was significant (*p* < 0.05) (Supplementary Figure S3D,E).

### 3.4 Gene Set Variation Analysis Function Enrichment Analysis

**FIGURE 2:**
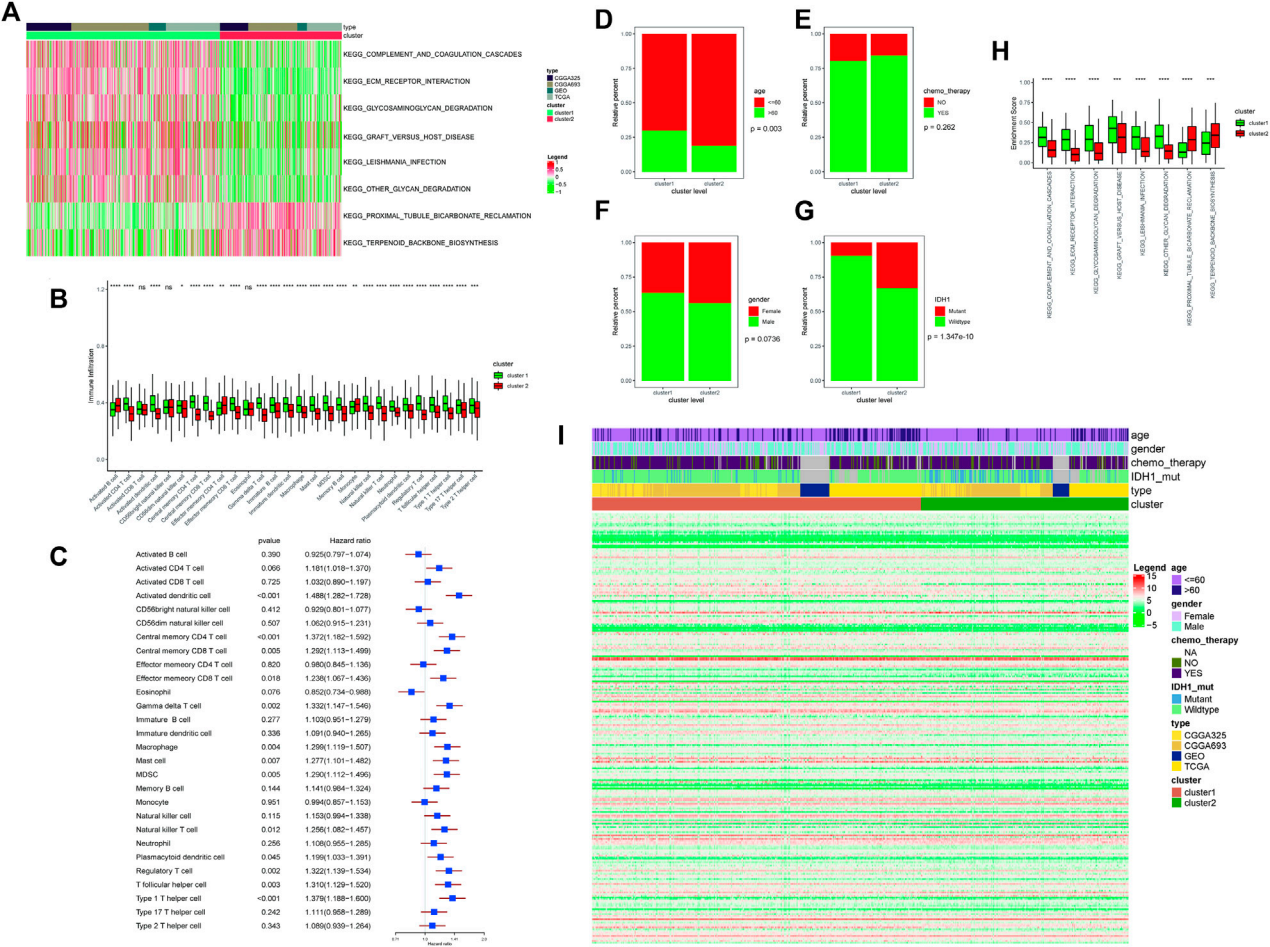
**(A)** Enrichment score results of differential enrichment items between the two subtypes. Legend column: different colors represent different scores; cluster column: green represents cluster 1, and red represents cluster 2; type column: different colors represent the names of different databases. **(B)** Differences in immune-infiltrating cells in different ferroptosis clusters; the abscissa axis represents different immune-infiltrating cells; the ordinate axis represents the degree of immune cell infiltration. Cluster column: green represents cluster 1, and red represents cluster 2; ^∗∗∗∗^means *p* < 0.0001, ^∗∗∗^means *p* < 0.001, ^∗∗^means *p* < 0.01, and ^∗^means *p* < 0.05. **(C)** Survival risk analysis of immune-infiltrating cells. The type of immune-infiltrating cells was listed on the left side; the HR and the forest plot corresponding to immune-infiltrating cells were listed on the right side. **(D)** Correlation analyses between ferroptosis clusters and clinical characteristics: the distribution of different age patients in cluster 1 and cluster 2. The abscissa axis represents different cluster levels; the ordinate axis represents relative percent. Age column: red means age≤ 60; green means age >60. **(E)** Correlation analyses between ferroptosis clusters and clinical characteristics: the distribution of patients receiving chemotherapy in cluster 1 and cluster 2. The abscissa axis represents different cluster levels; the ordinate axis represents relative percent. Chemotherapy column: red represents no chemotherapy, and green represents chemotherapy. **(F)** Correlation analyses between ferroptosis clusters and clinical characteristics: the distribution of patients’ gender ratio in cluster 1 and cluster 2. The abscissa axis represents different cluster levels; the ordinate axis represents relative percent. Gender column: red represents female, and green represents male. **(G)** Correlation analyses between ferroptosis clusters and clinical characteristics: the distribution of IDH1 mutation status in cluster 1 and cluster 2. The abscissa axis represents different cluster levels; the ordinate axis represents relative percent. IDH1 column: red represents mutant status, and green represents wild type. **(H)** Correlation analyses between ferroptosis clusters and clinical characteristics: the distribution of differential enrichment pathway scores in the two subtypes. The abscissa axis represents different signal pathways; the ordinate axis represents enrichment scores. Cluster column: green represents cluster 1, and red represents cluster 2. **(I)** Display of ferroptosis-related genes in different ferroptosis clusters. Age column: different colors represent different age ranges; gender column: different colors represent different genders; chemotherapy column: different colors represent the status of chemotherapy; IDH1 column: different colors represent IDH1 mutation status; type column: different colors represent different data types; cluster column: different colors represent different clusters.

GSVA was used to perform functional enrichment analysis on the samples, and then the R package “limma” was used to retrieve differential enrichment items between the two subtypes; eight enrichment items were obtained according to the threshold, and then a heatmap was drawn. Among them, KEGG_COMPLEMENT_AND_COAGULATION_CASCADES, KEGG_ECM_ RECEPTOR_INTERACTION, KEGG_GLYCOSAMINOGLYCAN_DEGRADATION, KEGG_ GRAFT_VERSUS_HOST_DISEASE, KEGG_LEISHMANIA_INFECTION, and KEGG_OTHER_GLYCAN_DEGRADATION were found to be enriched with higher scores in cluster 1, while KEGG_PROXIMAL_TUBULE_BICARBONATE_RECLAMATION and KEGG_TERPENOID_BACKBONE_BIOSYNTHESIS were found to be enriched with higher scores in cluster 2 (Figure 2A).

### 3.5 The Proportion and Difference of Immune-Infiltrating Cells in Different Ferroptosis Clusters Assessed by Single-Sample Gene Set Enrichment Analysis

The R package “GSVA” was used to calculate the enrichment score of 28 types of immune infiltration cells in cancer samples, and 25 types of immune infiltration cells were found to be significantly different between the two subtypes. Among them, activated CD4 T cell, activated dendritic cell, central memory CD4 T cell, effector memory CD8 T cell, gamma delta T cell, immature B cell, immature dendritic cell, macrophage, mast cell, MDSC, memory B cell, natural killer cell, natural killer T cell, neutrophil, plasmacytoid dendritic cell, regulatory T cell, T follicular helper cell, type 1 T helper cell, type 17 T helper cell, and type 2 T helper cell were found to be higher in cluster 1, while activated B cell, effector memory CD4 T cell, and monocyte were found to be higher in cluster 2 (Figure 2B).

After that, the univariate Cox regression analysis was performed on the proportion of immune cells, and the immune cells that were significantly related to the prognosis were screened with *p* < 0.05 as the threshold. Among them, activated dendritic cell, central memory CD4 T cell, central memory CD8 T cell, effector memory CD8 T cell, gamma delta T cell, macrophage, mast cell, MDSC, natural killer T cell, plasmacytoid dendritic cell, regulatory T cell, and T cells of both follicular helper cell and type 1 T helper cell were found to have a significant impact on the survival (Figure 2C).

### 3.6 The Correlation Analysis Between Different Ferroptosis Clusters and Clinical Characteristics

The proportions of age, gender, chemo_therapy, and IDH1 mutations in different subtypes would be calculated and plotted as a bar graph. Among them, only age was found to be significantly different between the two subtypes, and the remaining characteristics were not found to be significantly different. Between the two subtypes, seven of the eight differential enrichment pathway scores were found to be at a significantly different level, indicating that the two subtypes were closely related to the enrichment pathways (Figures 2D–H).

### 3.7 Display of Ferroptosis-Related Genes in Different “Ferroptosis Clusters”

The expression differences of ferroptosis-related genes among different “ferroptosis cluster” subtypes were counted and plotted as a heatmap (Figure 2I).

### 3.8 Screening for DEGs in “Ferroptosis Cluster” and Performing Enrichment Analysis for DEGs

The R package “limma” was used to screen for DEGs in different subtypes. Using |logFC|> 1 & FDR <0.05 as the thresholds, 491 DEGs were screened out, including 203 upregulated genes and 288 downregulated genes.

After that, GO and KEGG enrichment analyses of DEGs were performed. The GO enrichment analysis included three parts, namely: biological process (BP), cell component (CC), and molecular function (MF). Among them, the main pathways of BP enrichment were extracellular matrix organization and collagen fibril organization; the main pathways of CC enrichment were collagen-containing extracellular matrix and complex of collagen trimers; the main pathways of MF enrichment were extracellular matrix structural constituent, extracellular matrix structural constituent conferring tensile strength, and ion-gated channel activity. The main pathways of KEGG enrichment were ECM–receptor interaction and nicotine addiction. Relevant pathways were closely related to the ferroptosis process (Supplementary Figure S4).

### 3.9 “Ferroptosis.gene.cluster” Obtained by Cluster Analysis Based on DEGs

**FIGURE 3:**
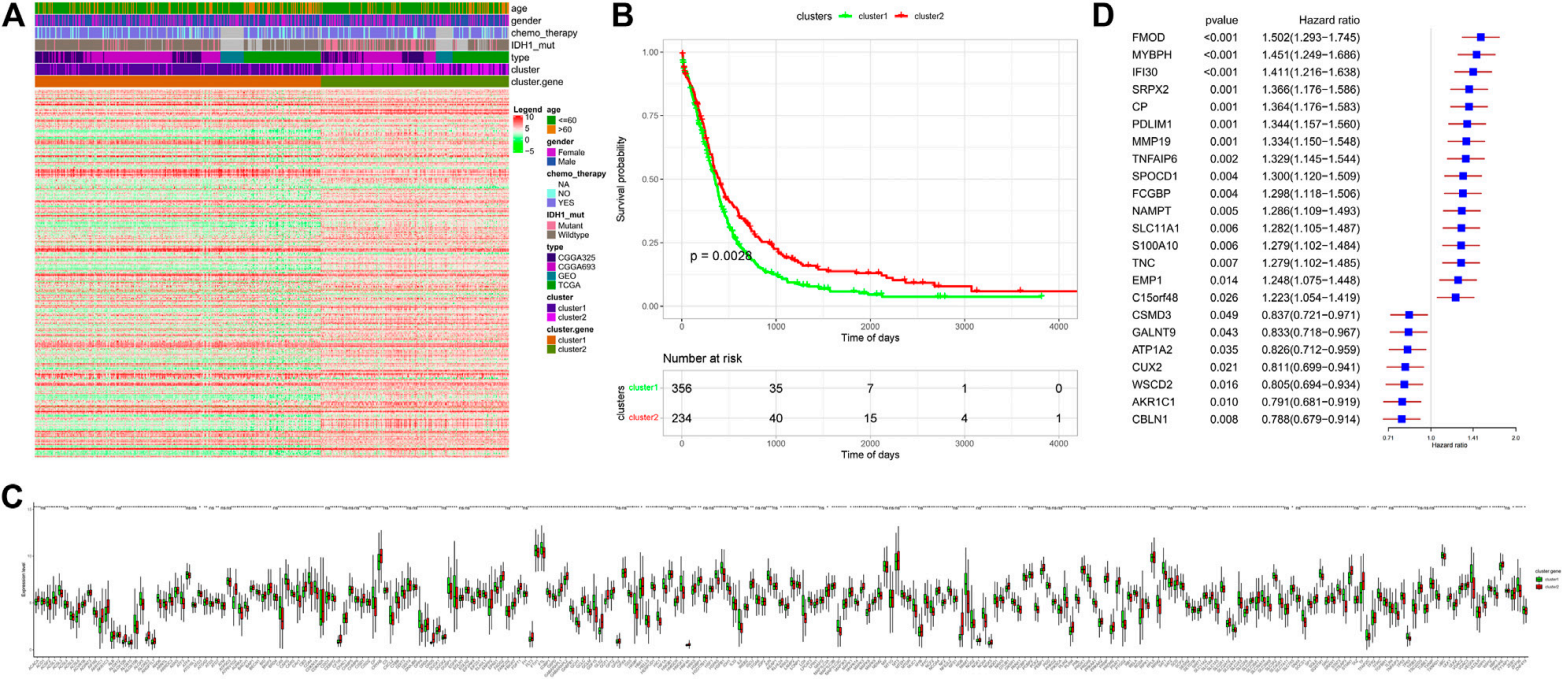
“Ferroptosis.gene.cluster” obtained by the clustering analysis of DEGs. **(A)** Heatmap of DEGs. Age column: different colors represent different age ranges; gender column: different colors represent different genders; chemotherapy column: different colors represent the status of chemotherapy; IDH1 column: different colors represent IDH1 mutation status; type column: different colors represent different data types; cluster column: different colors represent different clusters; Cluster.gene column: different colors represent different cluster.gene. **(B)** Kaplan–Meier survival analysis curve of Ferroptosis.gene.cluster grouping. The abscissa axis represents survival time, and the ordinate axis represents survival probability. **(C)** Expression differences of ferroptosis-related genes in the “Ferroptosis.gene.cluster” group. The abscissa axis represents the name of ferroptosis-related genes; the ordinate axis represents the expression level of the corresponding ferroptosis-related genes. **(D)** Risk score forest plot constructed by 23 key genes. The left column represents 23 key genes. The middle parts are *p*-value and hazard ratio. The right column is the forest plot of 23 key genes.

After unsupervised cluster analysis based on DEGs, two subtypes of “Ferroptosis.gene.cluster” were obtained, and the DEGs in different subtypes were displayed. Then, based on “Ferroptosis.gene.cluster” for survival analysis, the Kaplan–Meier survival curve was drawn, and the results showed that the survival curves of the two “Ferroptosis.gene.cluster” were significantly different. After that, the expression of ferroptosis-related genes in different “Ferroptosis.gene.cluster” was further analyzed, among which 196 ferroptosis-related genes were significantly different between the two “Ferroptosis.gene.clusters” (Figures 3A–C).

### 3.10 Construction of “Ferroptosis Gene Signatures” Based on the Risk Scores of DEGs in the “Ferroptosis Cluster”

Univariate Cox regression analysis was performed on the DEGs of different “ferroptosis clusters,” and 256 genes that were significantly related to the survival were screened out with *p* < 0.05 as the threshold. Then, the R package “randomForest” was used to perform random forest screening on genes that were significantly related to the survival, and 23 key genes (*FMOD, MYBPH, IFI30, SRPX2, CP, PDLIM1, MMP19, TNFAIP6, SPOCD1, FCGBP, NAMPT, SLC11A1, S100A10, TNC, EMP1, C15orf48, CSMD3, GALNT9, ATP1A2, CUX2, WSCD2, AKR1C1,* and *CBLN1*) were obtained with MeanDecreaseGini >0.72 as the threshold. Finally, based on the expression of these key genes, the PCA was performed on the samples, and the risk score of each sample was calculated (Figure 3D).

### 3.11 Prognostic Survival Assessment

**FIGURE 4:**
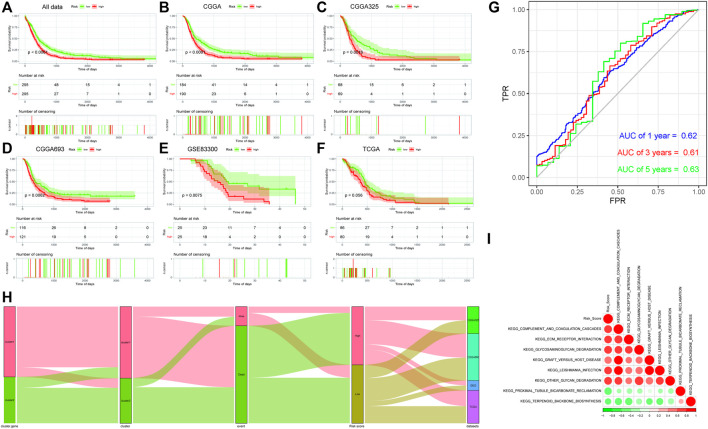
**(A–F)**: Kaplan–Meier survival analysis results of risk score subgroups in different data cohorts. The abscissa axis represents survival time, and the ordinate axis represents survival probability. **(A)** Kaplan–Meier survival analysis results of risk score subgroups in the comprehensive data cohort. **(B)** Kaplan–Meier survival analysis results of risk score subgroups in the CGGA data cohort. **(C)** Kaplan–Meier survival analysis results of risk score subgroups in the CGGA325 data cohort. **(D)** Kaplan–Meier survival analysis results of risk score subgroups in the CGGA693 data cohort. **(E)** Kaplan–Meier survival analysis results of risk score subgroups in the GSE83300 data cohort. **(F)** Kaplan–Meier survival analysis results of risk score subgroups in the TCGA data cohort. **(G)** Time-based ROC curve: the abscissa axis represents FPR (false-positive rate), and the ordinate axis represents TPR (true-positive rate). **(H)** Sankey diagram based on the distribution of characteristics. **(I)** Correlation analyses between the risk score and differential enrichment pathway score.

After the sample risk score was obtained, the samples were divided into high- and low-risk score groups by the median node, and then survival analysis was performed on the two groups, and then the Kaplan–Meier curve was drawn. The significant difference of the survival could be found between the two groups. At the same time, they were verified by the CGGA (CGGA325/CGGA693), GSE83300, and TCGA internal sub-data sets. Significant differences were also found in the sub-data sets [except the TCGA data set (*p* = 0.056)] of the two subtype samples (Figures 4A–F).

After that, the time-based ROC curve was drawn, and the AUC values of 1, 3, and 5 years were all greater than 0.6, indicating that the predictive model had a good predictive efficiency (Figure 4G). Finally, the Sankey diagram was drawn to indicate the relationship among data characteristics. Among them, cluster 1 of “ferroptosis clusters” had a higher proportion of deaths, and most of the survival samples had a lower risk, while the high- and low-risk samples were uniform from each sub-data set (Figure 4H).

### 3.12 Correlation Analysis of Risk Score, Ferroptosis-Related Genes, and Pathway Functions

The Pearson correlation coefficient between the risk score and the differential enrichment pathway scores was calculated. The results showed that most pathways were negatively correlated with the risk score, while they were positively correlated with each other. Risk score was negatively correlated with KEGG_PROXIMAL_TUBULE_BICARBONATE_RECLAMATION and KEGG_TERPENOID_BACKBONE_BIOSYNTHESIS pathways. In addition, the related pathway scores in cluster 1 were significantly lower than those of cluster 2, and the survival probability of samples in cluster 1 was lower, which was consistent with the risk score (Figure 4I).

**FIGURE 5:**
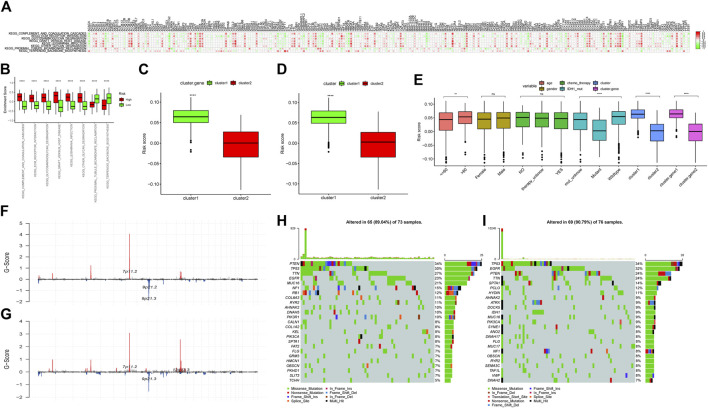
**(A)** Correlation analyses between ferroptosis-related genes and differential enrichment pathway scores. The abscissa axis represents the names of ferroptosis-related genes; the ordinate axis represents differential enrichment pathways. The legend on the right represents different Pearson correlation coefficients. **(B)** Difference in the enrichment scores of the subgroups with high- and low-risk scores. The abscissa axis represents pathways with different enrichment scores; the ordinate axis represents enrichment scores. Red represents the high-score group; green represents the low-score group. ^∗∗∗∗∗^means *p* < 0.0001; ^∗∗∗ ^means *p* < 0.001; ^∗∗^ means *p* < 0.01; and ^∗^ means *p* < 0.05. **(C)** Risk score difference analysis between the two “Ferroptosis.gene.cluster”. The abscissa axis represents different clusters; the ordinate axis represents risk score. ^∗∗∗∗^ means *p* < 0.0001; ^∗∗∗^ means *p* < 0.001; ^∗∗^ means *p* < 0.01; and ^∗^ means *p* < 0.05. **(D)** Risk score difference analysis between ferroptosis clusters. The abscissa axis represents different ferroptosis clusters; the ordinate axis represents different risk scores. ^∗∗∗∗^ means *p* < 0.0001; ^∗∗∗^ means *p* < 0.001; ^∗∗^ means *p* < 0.01; and ^∗^ means *p* < 0.05. **(E)** Risk score difference analysis for different clinical characteristics and different molecular types. The abscissa axis represents different clinical features and molecular types; the ordinate axis represents different risk scores. ^∗∗∗∗^means *p* < 0.0001; ^∗∗∗^ means *p* < 0.001; ^∗∗^means *p* < 0.01; and ^∗^ means *p* < 0.05. **(F)** Difference of CNV sites in the high-risk score group. The abscissa axis represents the location of CNV on the chromosome; the ordinate axis represents G-score. **(G)** Difference of CNV sites in the low-risk score group. The abscissa axis represents the location of CNV on the chromosome; the ordinate axis represents G-score. **(H)** Mutations in the high-risk score group. The left column represents the name of the mutant genes; the right column represents the percentage of genes with mutations; different colors represent different mutation types. **(I)** Mutations in the low-risk score group. The left column represents the name of the mutant genes; the right column represents the percentage of genes with mutations; different colors represent different mutation types.

The Pearson correlation coefficient between the ferroptosis-related genes and the differential enrichment pathway scores was further calculated and visualized by the R package “corrplot” (Figure 5A).

### 3.13 Differential Analysis of the Risk Score in Different Groups

#### 3.13.1 Differential Enrichment Analysis Scores Between High- and Low-Risk Score Groups

By analyzing the differential enrichment analysis scores between the high- and low-risk score groups, it was found that eight pathways were significantly different between the high- and low-risk groups. The first six pathways in the low-risk group had significantly higher scores than those of the high-risk group. However, the scores of KEGG_PROXIMAL_TUBULE_BICARBONATE_RECLAMATION and KEGG_TERPENOID_BACKBONE_BIOSYNTHESIS in the high-risk group were significantly higher than those of the low-risk group, which was consistent with the results of the previous analysis (Figure 5B).

#### 3.13.2 Differential Analysis of the Risk Score in Different “Ferroptosis.gene.clusters”

By analyzing the risk score differences in different “Ferroptosis.gene.clusters,” it was found that the risk score in gene.cluster 1 was significantly higher than that of gene.cluster 2 (Figure 5C), and the relevant conclusions were consistent with the previous analysis.

#### 3.13.3 Differential Analysis of the Risk Score in Different “Ferroptosis Clusters”

Through differential analysis of the risk score in different “Ferroptosis clusters,” it was indicated that the risk score in cluster 1 was significantly higher than that of cluster 2 (Figure 5D), and the relevant conclusions were consistent with the previous analysis.

### 3.14 Differential Analysis of the Risk Score in Clinical Features and Different Molecular Types

The distribution of different risk scores in age, gender, chemo_therapy, IDH1 mutation grouping, “ferroptosis cluster,” and “Ferroptosis.gene.cluster” was further checked and plotted as box plots. The results showed that the distributions of different risk scores in age, IDH1 mutation group, “ferroptosis cluster,” and “Ferroptosis.gene.cluster” were significantly different (Figure 5E).

### 3.15 Correlation Analyses Between Risk Score and Tumor Mutation Burden, homologous recombination deficiency, Neoantigen Load, Chromosomal Instability, and mRNAsi

The correlations between risk score and TMB, HRD, neoantigen load, chromosomal instability, and mRNAsi were calculated, and then a linear correlation graph was drawn. The results indicated that risk score had a strong correlation with mRNAsi (R = −0.498), which was consistent with the previous report (Malta et al., 2018). In addition, similar significant correlation could also be found between the risk score and HRD, HRD_TAI, and HRD_LST (Supplementary Figure S5).

### 3.16 The Landscape of High- and Low-Risk Score Groups

The differences of CNV sites between the high- and low-risk score groups were checked, and it was found that the CNV sites of the two groups were similar. However, the G-score of 7p11.2 in the high-risk group was slightly higher than that of the low-risk group, indicating that the proportion of the relevant sites was higher in the high-risk group. In addition, 9p21.2 and 9p21.3 in the high-risk group also had higher G-scores, while 12q13.3 in the low-risk group had a higher G-score (Figures 5F,G).

Then, combined with risk score grouping, the genetic mutations of the two group samples would be displayed. The waterfall chart showed that both of the two risk groups had higher mutation rates. Among them, PTEN had the highest mutation rate in the high-risk group, while TP53 enjoyed a higher mutation rate in the low-risk group (Figures 5H,I).

### 3.17 Immunotherapy Analysis Results in the High- and Low-Risk Score Groups (TIDE Prediction + GDSC)

#### 3.17.1 Analysis Results of GDSC in the High- and Low-Risk Groups, Estimated by the IC_50_ Value

The drug treatment response of the high- and low-risk group samples was further analyzed. The results indicated that the two risk groups enjoyed significant differences in the efficacy of cisplatin, vinblastine, gemcitabine, and paclitaxel. Moreover, the samples in the low-risk group were much more sensitive to cisplatin, vinblastine, gemcitabine, and paclitaxel.

After that, the TIDE score was used to predict the immunotherapy efficacy. The results indicated that the TIDE score of the high-risk group was significantly higher than that of the low-risk group, implying that the high-risk group might have a poorer immunotherapy efficacy (Supplementary Figure S6A-E).

#### 3.17.2 Expression Differences of Immune Check Sites in the High- and Low-Risk Groups

The expression of immune checkpoints in the high- and low-risk groups was checked. The results indicated that the expression of CD274, CTLA4, PDCD1, and LAG3 in the samples of the high-risk group was significantly higher than that of the low-risk group, suggesting that the high expression of relevant genes might be one of the reasons that affected GBM treatment efficacy (Supplementary Figure S6F–I).

### 3.18 Immunohistochemical Validation Results of Clinical Samples

**FIGURE 6:**
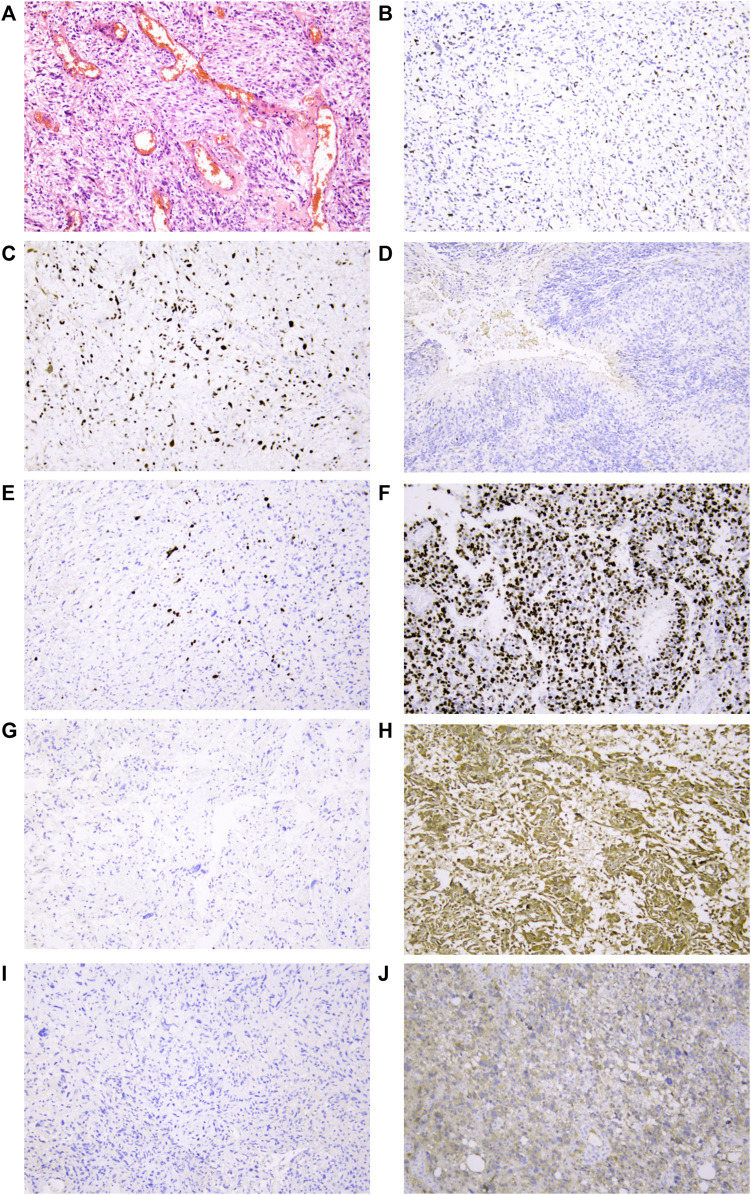
Representative IHC analyses of p53/Ki-67/MGMT/IDH1R132H protein expression in cancer cells of glioblastoma patients. **(A)** Representative glioblastoma with HE staining. **(B)** Normal/wild-type p53 protein expression pattern with partly and weakly positive expression in tumor nuclei. Two patterns were identified as abnormal/mutant-staining pattern. **(C)** Abnormal overexpression of p53 protein with strong staining in nearly all tumor nuclei compared to internal control central fibroblasts. **(D)**. Abnormal complete absence of p53 staining with sufficient staining of internal controls (fibroblasts, endothelial cells, or lymphocytes). **(E)** Low proportion of Ki-67 protein expression in tumor nuclei suggested that the tumor has low proliferative activity. **(F)** High proportion of Ki-67 protein expression in tumor nuclei suggested that the tumor has high proliferative activity. **(G)** Negative expression of MGMT protein in tumor nuclei might be related to MGMT methylation. **(H)** Strong positive expression of MGMT protein in tumor nuclei. **(I)** IDH1 R132H wild-type protein expression pattern with cytoplasmic negative staining of tumor cells. **(J)** IDH1 R132H mutation protein expression pattern with cytoplasmic positive staining of tumor cells. All mages were taken at 10 ×10 magnification on the Leica DM2000 microscope.

From forty-two glioblastoma patients, including 17 females and 25 males, histopathological sections were collected. The basic characteristics of all enrolled clinical samples are summarized in Table 2. There were 15 patients with *P53* gene mutation, and 27 patients were *P53* wild-type. There was no significant difference in the P53 status between the two parts. *IDH1* gene mutation was detected in six patients, while *Ki-67* > 30% was found in 14 patients. Typical immunohistochemical staining results, including *P53, Ki-67, MGMT*, and *IDH1*, are provided in Figure 6.

**TABLE 2:** Basic characteristics of all enrolled clinical samples.

Characteristic	P53 Wild-type	P53 mutation	*p*-value
N	27	15	-
Age, mean ± SD	57.89 ± 11.14	59.93 ± 9.92	0.557
Gender, n (%)	-	-	0.708
Female	12 (28.6%)	5 (11.9%)	-
Male	15 (35.7%)	10 (23.8%)	-
IDH1 mutation, n (%)	-	-	0.649
Wild-type	24 (57.1%)	12 (28.6%)	-
Mutation	3 (7.1%)	3 (7.1%)	-
Ki-67 expression, n (%)	-	-	0.085
≤30%	21 (50%)	7 (16.7%)	-
-	6 (14.3%)	8 (19%)	-
MGMT expression, n (%)	-	-	0.740
Low or negative	14 (35.9%)	10 (25.6%)	-
Positive	10 (25.6%)	5 (12.8%)	-

## 4.Discussion

The flow diagram of the study is provided in Supplementary Figure S1. Among the 257 ferroptosis-related genes with expression information, there were 239 ferroptosis-related genes whose expression was significantly different between cancer and normal samples (*p* < 0.05), indicating that the expression of most ferroptosis-related genes was related to glioblastoma (Supplementary Figure S2). The results of PCA also further verified the aforementioned conclusions (Supplementary Figure S3A,B). Some of those genes, such as *CDKN2A* (Kraus et al., 2001; Hu et al., 2017), *CA9* (Said et al., 2007), and *HSPB1* (Rajesh et al., 2020), were also found in previous reports, which further confirmed the feasibility of our study design. Further analysis found that *CDKN2A, TP53, IDH1, and EGFR* were still the most abnormally altered genes in glioblastoma (Figure 1) (Kraus et al., 2001; Hu et al., 2017; Hu et al., 2022; Hu et al., 2018), while the most common variation in SNP was C > T (Figure 1C) (McDonald et al., 2013; Mistry et al., 2018). This was similar to the mutation frequency of *TP53* and *IDH1* obtained in the immunohistochemistry of clinical samples (Table 2; Figure 6). PCA also showed that there were significant differences in ferroptosis-related genes between cancer and normal tissues (Supplementary Figure S3B). This further confirmed the important role of ferroptosis in glioblastoma.

Clustering analysis is a commonly used method for analyzing abnormal genome alterations in research (Töpfer et al., 2017; Adolfsson et al., 2019; Raymaekers and Zamar, 2020). Based on the expression information of ferroptosis-related genes in cancer samples, unsupervised clustering analysis was carried out. It was found that positive correlation was the main trend among ferroptosis-related genes (Supplementary Figure S3C), which was rarely mentioned in glioblastoma before. Through survival analysis of the two clusters obtained by the unsupervised clustering analysis of cancer samples, it was found that there was a statistically significant difference in the survival curve (Supplementary Figure S3D,E); similar differences could also be seen when GSVA, ssGSEA, and correlation analysis of clinical characteristics were completed (Figures 2A–H). Further analysis indicated that 12 types of immune cells had significant effects on survival (Figure 2C). Those would be helpful for us to use them to evaluate the clinical survival prognosis (Thomas et al., 2015; Du Four et al., 2016; Vinuesa et al., 2016; Pellegatta et al., 2018; Bayik et al., 2020; Han et al., 2020; Lupo and Matosevic, 2020; Xiong et al., 2020; Zhou et al., 2021; Campian et al., 2022).

After the differential expression analysis of ferroptosis-related genes in different ferroptosis clusters were carried out, it was found that the mutation frequency of *IDH1* in cluster 2 was significantly higher than that of cluster 1 (Figures 2G–I), which further explained the reason for that cluster 2 had better survival prognosis than cluster 1 (Supplementary Figure S3E). It also further affirmed the potential prognostic significance of *IDH1* mutation in glioblastoma (Nobusawa et al., 2009; Yan et al., 2009; Molenaar et al., 2014), which was also consistent with our focus on *IDH1* in clinical works (Table 2; Figure 6).

Both Gene Ontology (GO) and KEGG enrichment analyses of DEGs in ferroptosis clusters suggested that ferroptosis played an important role at a certain time in the entire process of glioblastoma (Supplementary Figure S4) (Ma et al., 2019; Hynes and Naba, 2012; Bryukhovetskiy et al., 2019; Kiyokawa et al., 2021). Some of them, such as ECM–receptor interaction, nicotine addiction, and complex of collagen trimers, were rarely reported in glioblastoma (Bryukhovetskiy et al., 2019), which might provide some new ideas for further research. “Ferroptosis.gene.cluster” obtained by clustering analysis based on DEGs also showed similar prognostic effect as before (Supplementary Figure S3D,E, and Figure 3). Furthermore, the differences presented between Ferroptosis.gene.cluster 1 and Ferroptosis.gene.cluster 2 in the DEG heatmap (Figure 3A) were much more pronounced than previous clusters (Figure 2I). Statistically significant expression differences could also be found in most of the “Ferroptosis.gene.cluster” groups (Figure 3C). This means that this kind of clustering method may be a much more better approach to reveal abnormal information in glioblastoma. This was the basis for the subsequent construction of the risk score predictive model containing 23 genes. Therefore, we believed that the 23-gene risk scoring model (*CP, EMP1, AKR1C1, FMOD, MYBPH, IFI30, SRPX2, PDLIM1, MMP19, SPOCD1, FCGBP, NAMPT, SLC11A1, S100A10, TNC, CSMD3, ATP1A2, CUX2, GALNT9, TNFAIP6, C15orf48, WSCD2,* and *CBLN1*) constructed based on this might have a better prognostic prediction efficacy (Xiao et al., 2021; Yu et al., 2022), which had been verified in the subsequent analysis results (Figures 4A–G). Also, this prognostic prediction advantage was particularly evident when it was depicted in the form of “risk score” in the Sankey diagram (Figure 4H).

For those 23 genes, 3 (*CP, EMP1*, and *AKR1C1*) of them were found to be reported in ferroptosis-related studies (Yang et al., 2019; Huang et al., 2021; Huang et al., 2021), while 19 genes (*CP, EMP1, AKR1C1, FMOD, MYBPH, IFI30, SRPX2, PDLIM1, MMP19, SPOCD1, FCGBP, NAMPT, SLC11A1, S100A10, TNC, CSMD3, ATP1A2, CUX2,* and *GALNT9*) were previously reported in glioblastoma. The four tumor-related genes (*TNFAIP6, C15orf48, WSCD2*, and *CBLN1*) were neither reported in ferroptosis-related reports nor in glioblastoma-related studies (Wei et al., 2012; Su et al., 2013; Shin et al., 2020; Bushel et al., 2022), which were first found by us. While there was not necessarily a causal relationship between related things, these findings at least provided a range of options for further investigation. Therefore, the specific roles of these genes in glioblastoma still needed to be further verified by basic experiments.

We enrolled 23 genes in this risk score prediction model, which could minimize the bias in the prediction results due to the inclusion of too few predictive genes. Subsequent analysis showed that most of the pathways were negatively correlated with risk score, while these pathways were positively correlated with each other (Figure 4I). The risk score of related pathways in cluster 1 was significantly lower than that in cluster 2, and the survival probability of cluster 1 was lower, which was consistent with the results of risk score-related analysis results (Supplementary Figure S3D–3E Figure 2A, Figure 4, Figures 5A–D). This further verified the consistent trend of our overall analysis results and the feasibility of the analysis method. In the follow-up analysis results, it was found that the distribution of risk score was significantly different among age, IDH1 mutation group, “ferroptosis cluster,” and “Ferroptosis.gene.cluster” (Figure 5E). This will provide an important reference for comprehensively judging the prognosis of patients in our clinical work.

Through the correlation analysis between risk score and TMB, homologous recombination deficiency (HRD), neoantigen load and chromosomal instability, and mRNAsi (Supplementary Figure S5), we found that it had a significant correlation with HRD, HRD_TAI, and HRD_LST, especially with mRNAsi (R = −0.498), which would help us apply the mRNAsi to single-cell data to reveal patterns of intratumoral molecular heterogeneity, leading to a better understanding of glioblastoma (Malta et al., 2018; Zheng et al., 2021). By analyzing the differences of CNV loci in the high- and low-risk score groups, it was found that a G-score of 12q13.3 (mainly including *OS9* gene, *CDK4* gene, and *SAS* gene) in the low-risk group was higher (Figures 5F,G), which was considered to be the characteristic locus (Reifenberger et al., 1994; Rollbrocker et al., 1996). Regardless of grouping, missense mutations, including PTEN, TP53, EGFR, and TTN, consistent with former reports and clinical verification (Table 2 and Figure 6), were still the predominant genomic alteration type in glioblastoma (Figures 5H,I) (Smith et al., 2001; Ohgaki et al., 2004; Lee et al., 2006; Binder et al., 2018). Among them, PTEN had the highest alteration rate in the high-risk score group, while TP53 had a higher alteration rate in the low-risk score group (Figures 5H,I).

TIDE and GDSC assays were often used to assess anti-tumor therapy responses (Wang et al., 2021; Cascio et al., 2021; Cancer Cell Line Encyclopedia Consortium, 2015). In this study, the results of these two analyses showed significant differences in different risk score subgroups (Supplementary Figure S6A–E), further supporting the feasibility of the risk score model to be used for the clinical prognostic assessment. Subsequent immune checkpoint analyses revealed that the expressions of CD274, CTLA4, PDCD1, and LAG3 in the high-risk group were significantly higher than those of the low-risk group, suggesting that the high expression of related genes might be one of the reasons that affected GBM treatment and prognosis (Supplementary Figure 6F–I), which was also consistent with previous reports on the correlation between those four genes and the glioblastoma prognosis (Andrews et al., 2020; Du et al., 2020; Yang et al., 2021; Bi et al., 2022; Preddy et al., 2022).

There were three groups of consistent clustering analyses in this study. The first was the gene clustering analysis for Ferroptosis gene; the second was the clustering analysis based on the expression of Ferroptosis gene, named “Ferroptosis.cluster”; the last was the expression of DEGs based on Ferroptosis.cluster named “Ferroptosis.gene.cluster.” The whole process of cluster analysis was also an optimization process of all the data. Therefore, the consistency of the obtained results had a better feasibility and reliability. In addition, the data size of this study was relatively sufficient, and the analysis results of the verification data were consistent from each other and had a statistical significance, which might provide a certain reference for later basic or clinical research in this field.

## 5 Conclusion

In glioblastoma, there were a large number of abnormal ferroptosis-related alterations, which were significant for the prognosis of patients. The risk score-predicting model including 23 ferroptosis-related genes might have a better predictive significance.

## Data Availability

The datasets presented in this study can be found in online repositories. The names of the repository/repositories and accession number(s) can be found in the article/Supplementary Material.

## Abbreviations

TMB, tumor mutational burden; PCA, principal component analysis; GSVA, gene set variation analysis; ssGSEA, single-sample gene set enrichment analysis; FPR, false-positive rate; TPR, true-positive rate; HRD, homologous recombination deficiency; TAI, telomeric–allelic imbalance; LST, large-scale state transitions; LOH, loss of heterozygosity; CNV, copy number variation; TIDE, tumor immune dysfunction and exclusion; GDSC, Genomics of Drug Sensitivity in Cancer; MSigDB, Molecular Signatures Database.
