# Supplementary figures and images for: Comprehensive Analyses of Ferroptosis-Related Alterations and Their Prognostic Significance in Glioblastoma
Source: Front Mol Biosci. 2022 Jun 3;9:904098. doi: 10.3389/fmolb.2022.904098 (PMC9204216; doi:10.3389/fmolb.2022.904098)

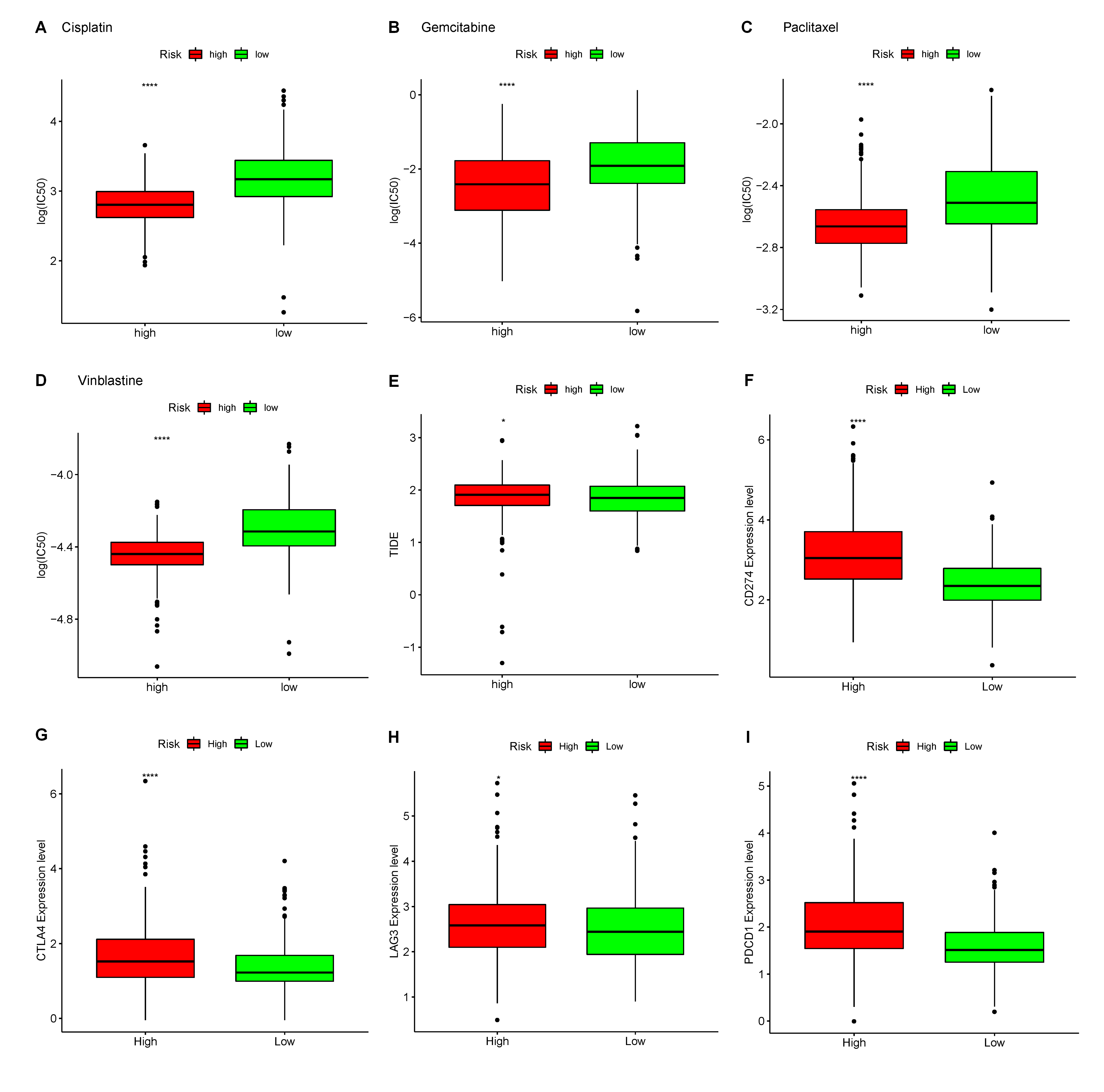

Supplement: Supplementary file 1 [file Image6.tif]

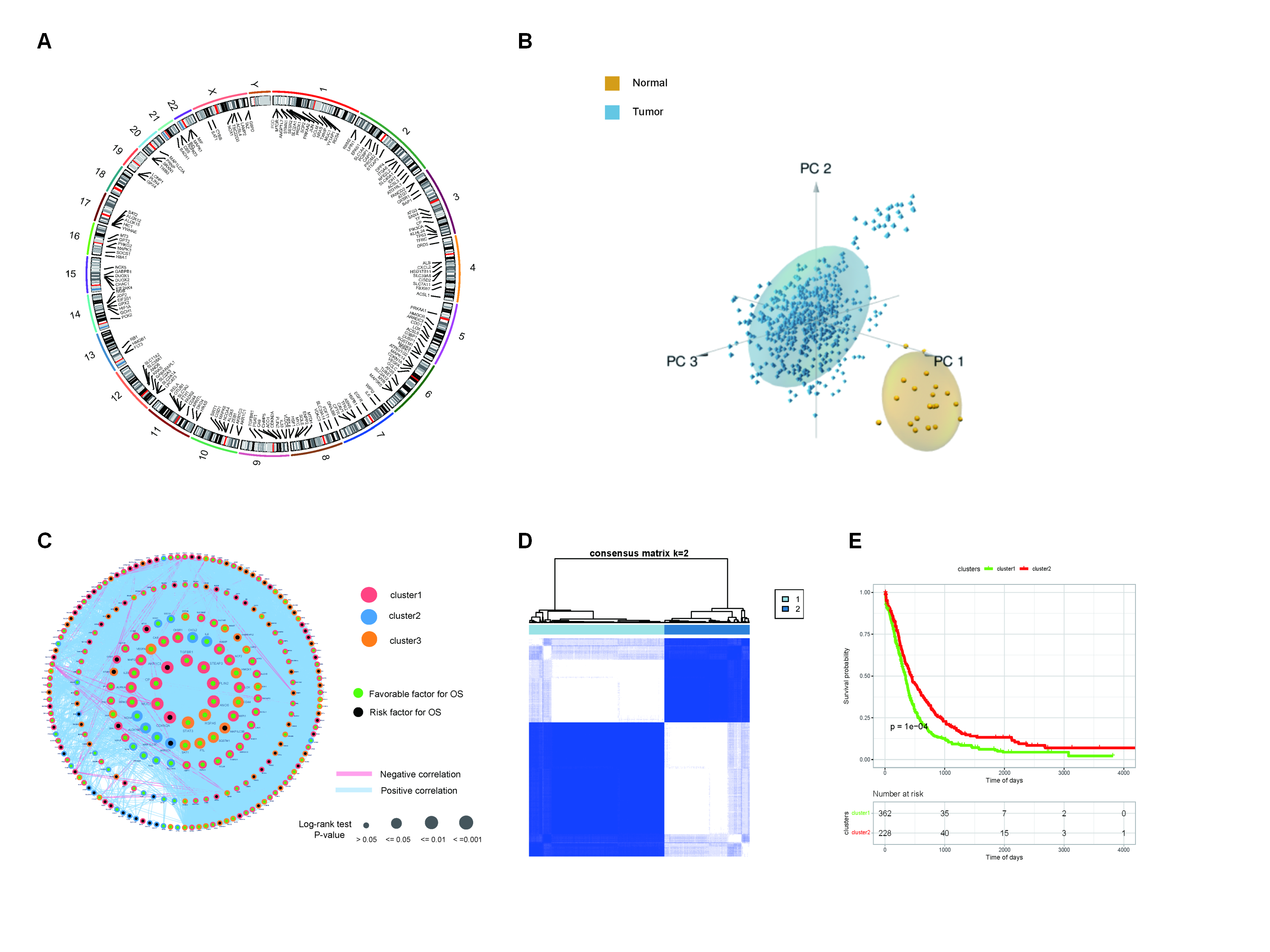

Supplement: Supplementary file 2 [file Image3.tif]

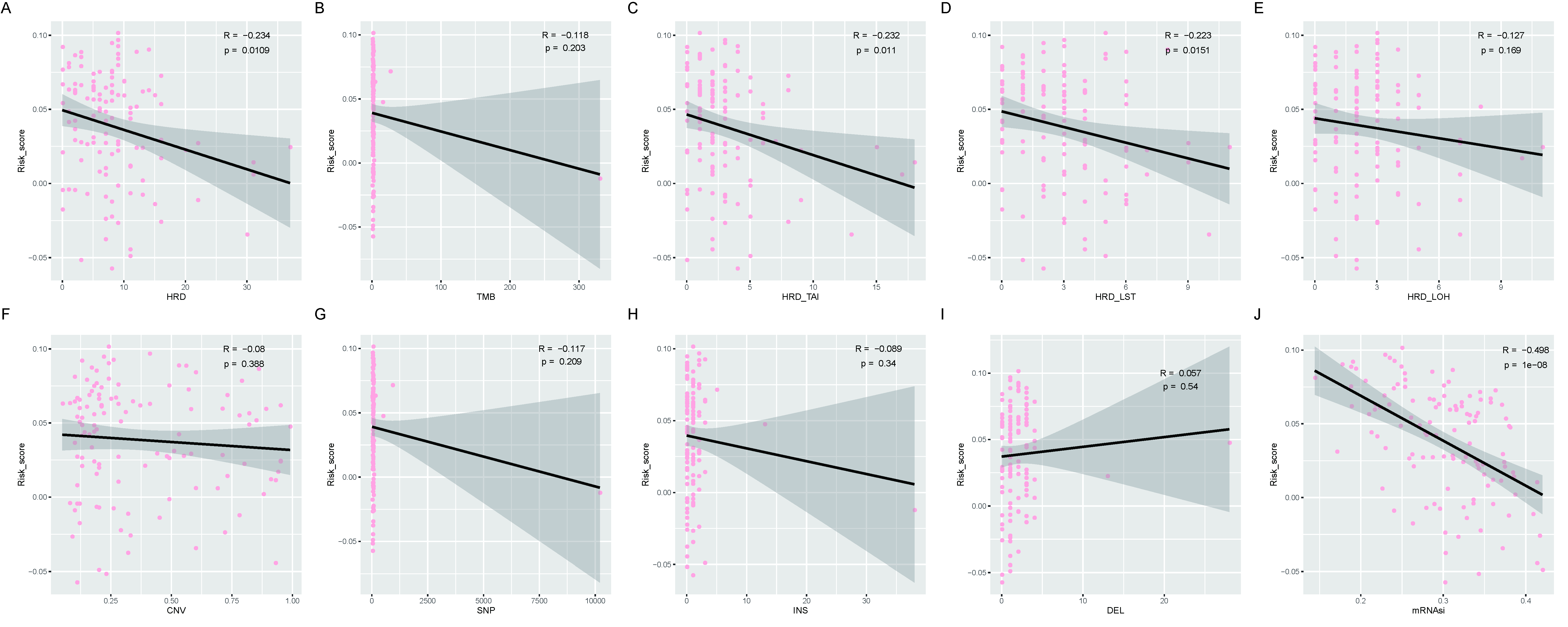

Supplement: Supplementary file 3 [file Image5.tiff]

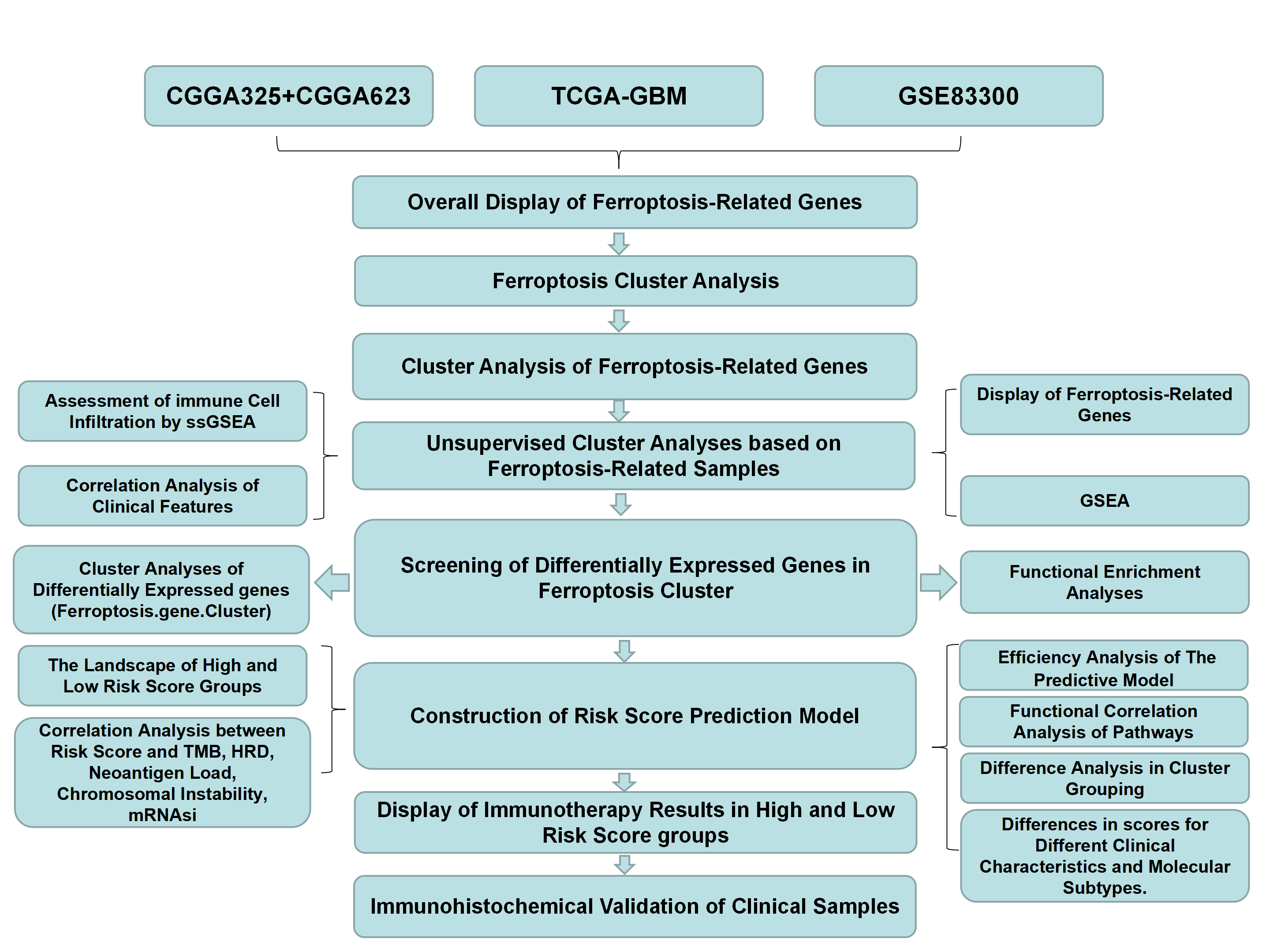

Supplement: Supplementary file 4 [file Image1.tif]

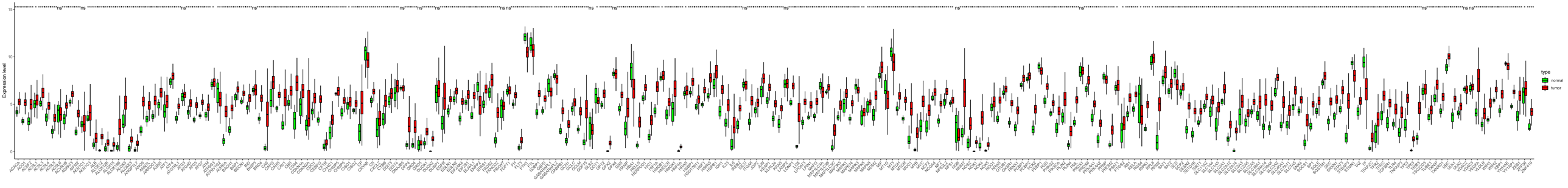

Supplement: Supplementary file 5 [file Image2.tiff]

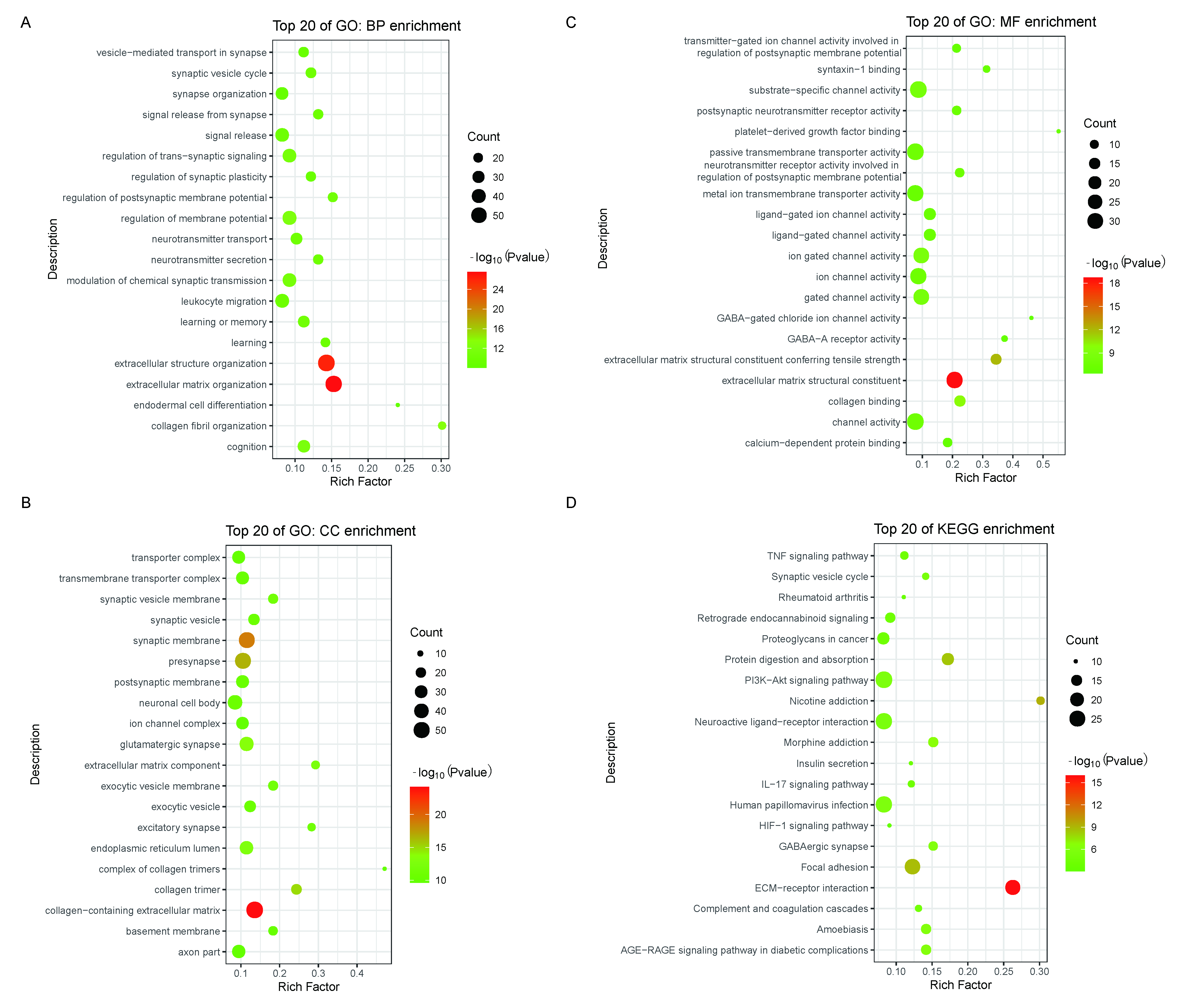

Supplement: Supplementary file 6 [file Image4.tiff]
